# Characteristics of Adult Sepsis Patients in the Intensive Care Units in a Tertiary Hospital in Jordan: An Observational Study

**DOI:** 10.1155/2021/2741271

**Published:** 2021-12-30

**Authors:** Anas H. A. Abu-Humaidan, Fatima M. Ahmad, Maysaa' A. Al-Binni, Amjad Bani Hani, Mahmoud Abu Abeeleh

**Affiliations:** ^1^Department of Pathology, Microbiology, and Forensic Medicine, School of Medicine, The University of Jordan, Amman, Jordan; ^2^Department of Clinical Sciences, School of Science, The University of Jordan, Amman, Jordan; ^3^Department of General Surgery, Section of Cardiovascular Surgery, Jordan University Hospital, Amman, Jordan

## Abstract

Sepsis is a global health issue that is commonly encountered in the intensive care unit (ICU) and is associated with high morbidity and mortality. Available data regarding sepsis in low- and middle-income countries (LMIC) is lacking compared to higher income countries, especially using updated sepsis definitions. The lack of recent data on sepsis in Jordan prompted us to investigate the burden of sepsis among Jordanian ICU patients. We conducted a prospective cohort study at Jordan University Hospital, a tertiary teaching hospital in the capital, Amman. All adult patients admitted to the adult ICUs between June 2020 and January 2021 were included in the study. Patients' clinical and demographic data, comorbidities, ICU length of stay (LOS), medical interventions, microbiological findings, and mortality rate were studied. Descriptive and inferential statistics were used to analyse data from patients with and without sepsis. We observed 194 ICU patients during the study period; 45 patients (23.3%) were diagnosed with sepsis using the Sepsis-3 criteria. Mortality rate and median ICU LOS in patients who had sepsis were significantly higher than those in other ICU patients (mortality rate, 57.8% vs. 6.0%, *p* value < 0.001, resp., and LOS 7 days vs. 4 days, *p* value < 0.001, resp.). Additionally, sepsis patients had a higher combined number of comorbidities (2.27 ± 1.51 vs. 1.27 ± 1.09, *p* value < 0.001). The use of mechanical ventilation, endotracheal intubation, and blood transfusions were all significantly more common among sepsis patients. A causative organism was isolated in 68.4% of sepsis patients with a prevalence of Gram-negative bacteria in 77.1% of cases. While the occurrence of sepsis in the ICU in Jordan is comparable to other regions in the world, the mortality rate of sepsis patients in the ICU remains high. Further studies from LMIC are required to reveal the true burden of sepsis globally.

## 1. Introduction

Sepsis is commonly encountered in the intensive care unit (ICU). An audit of ICU patients across several continents identified nearly 30% of patients as having sepsis during their ICU stay [1], with rates varying among studies and regions [2, 3]. Mortality rates associated with sepsis vary by region as well but are consistently high, especially in the elderly [4]. The aging population around the world led to the recognition of sepsis as an important cause of mortality [5].

Studies discussing the rate and demographics of sepsis are often carried out in high-income countries [6, 7]; such studies form the basis for patient management guidelines. More studies from low- and middle-income countries (LMIC) can shed light on the difficulties faced in sepsis management in resource challenged environments and would better reflect the true global burden of sepsis [8, 9].

Sepsis is associated with several pathological and biochemical disturbances. Consequently, many definitions for sepsis have been put forth. *The Third International Consensus Definition for Sepsis and Septic Shock* (Sepsis-3) considered sepsis as life-threatening organ dysfunction caused by a dysregulated host response to infection [10]. Consistency in definition can offer a better interpretation of epidemiological studies and recognition of disease burden. In Jordan, there are no studies on the burden and characteristics of adult sepsis patients using the Sepsis-3 definition.

This prospective cohort study conducted over the period between July 2020 and January 2021 examined all adult patients admitted to the adult ICUs in a tertiary academic hospital in the Jordanian capital Amman to achieve two goals: first, to understand the burden of critical illness and sepsis by reporting on length of stay, comorbidities, medical interventions, and mortality rates of ICU patients; second, to assess the microbiological findings in sepsis patients.

## 2. Materials and Methods

### 2.1. Study Design and Population

This was a single-centre prospective cohort study conducted at the adult ICUs of Jordan University Hospital (JUH), Amman, the largest academic tertiary hospital in the capital. JUH serves over 500000 patients from various regions of the country in outpatient clinics each year. We followed up 194 patients admitted to JUH ICUs over a period of 6 months (from July 15, 2020, through January 15, 2021). Inclusion criteria included all patients who were ≥18 years of age admitted to JUH's adult ICUs within the study period. The only exclusion criterion was age under 18 years. The total bed capacity of JUH is about 600, with approximately 32 beds distributed to 3 adult ICUs: surgical (SICU), medical (MICU), and anesthesia (AICU) ICUs. In 2020, these ICUs experienced 635 admissions.

The study population was classified into two groups, including patients having sepsis anytime during their ICU stay period and those who did not. The diagnosis of sepsis was based on the diagnostic guidelines of Sepsis-3 that were set in 2016 by the *Third International Consensus Definitions for Sepsis and Septic Shock* [10]. Sepsis was defined as a suspected or documented infection plus an acute organ dysfunction represented by an increase in Sequential Organ Failure Assessment (SOFA) score equal to or greater than 2 points [10, 11]. ICU mortality was defined as death during anytime of the ICU stay. ICU length of stay (LOS) was calculated as the date of ICU discharge−the date of ICU admission. Hospital LOS was calculated as the date of hospital discharge−the date of hospital admission.

### 2.2. Ethical Approval

The study protocol was approved by the Institutional Review Board (IRB) at JUH (Ref. no. 189/2020). In addition, the work was conducted according to the principles of Good Clinical Practice (GCP) that has its origin in the Declaration of Helsinki (64th World Medical Association General Assembly, Fortaleza, Brazil, October 2013). All collected data were treated with confidentiality.

Participation in the study was voluntary. Following a full explanation of the study objectives, a written and signed informed consent was obtained from all conscious patients who agreed to participate. Assessing the level of consciousness involved checking orientation: participants who were able to promptly and spontaneously state their name, location, and date or time were said to be conscious [12]. For patients who were unconscious or unable to consent at the time of admission, consent was obtained from first-degree relatives. However, consent was sought from those who survived once they regained consciousness or improved clinically to a stage where they can consent.

### 2.3. Data Collection

For each patient, the recorded data were categorised into demographic, clinical, and laboratory variables. Demographic variables included age, sex, height, weight, smoking status, comorbidities, date of admission to the hospital or ICU, and date of discharge from the hospital or ICU.

Clinical variables included ICU section (divided into SICU, MICU, and AICU), source of ICU admission (operation room, hospital wards, emergency department, etc.), reasons for admission, suspected origin of infection for patients with sepsis, vital signs on admission, and medical interventions (mechanical ventilation, catheterization, and blood transfusion).

Laboratory variables included haemoglobin, packed cell volume (PCV), total WBC count, neutrophil count, lymphocyte count, platelet count, creatinine, random blood sugar (RBS), and electrolytes (Na, K, and CL), as well as microbiological findings such as culture results and type of samples used for culture.

### 2.4. Data Analysis

Data are presented as count or percentages or both for categorical variables, and as mean ± standard deviation with or without the median for continuous variables. To compare measurements of sepsis and nonsepsis groups, as well as survivors and nonsurvivors, the unpaired *T* test (UT), Fisher's exact test (FE), or Mann–Whitney *U* test (MW) was used when appropriate, and Shapiro-Wilk test was used to test the normality distribution of data. Data generated was organised in Microsoft Excel and statistical analysis was carried out using IBM Statistical Package for the Social Sciences (SPSS) version 25.0 (USA) and GraphPad prism 8 (USA).

## 3. Results

### 3.1. Characteristics and Outcome of the Study Cohort

We analysed the demographic and clinical data of 194 patients admitted to the adult ICUs at JUH during the study period. To observe factors associated with ICU survival, we divided the cohort into patients discharged alive from the ICU (survivors) and patients who died during their ICU stay (nonsurvivors). The average age in the cohort was 60 ± 16 years, with 107 (55.2%) males and 87 (44.8%) females (Table 1). Most of the ICU patients had at least 1 comorbidity (76.8%). The most commonly encountered comorbidity was diabetes in 93 patients (47.9%). Nonsurvivors were significantly more likely to suffer from renal disease and chronic obstructive pulmonary disease (COPD) compared to survivors (48.6% vs. 14.5%, *p* < 0.001, and 20.0% vs. 5.0%, *p* = 0.008, resp.) (Table 1). Moreover, hypertension was common in this cohort (57.2%) but was not significantly more common in nonsurvivors (Table 1). Additionally, vital signs were recorded for all ICU patients on admission and can be found in Supplementary Table 1.

When assessing factors related to ICU admission and stay, the majority of patients were found to have been admitted either through the emergency department (42.3%) or the surgical ward (39.2%), and the majority were serviced in the surgical ICU (63.9%). Patients admitted to the MICU formed a small part of the study cohort (14, 14.2%) but had the highest percentage of nonsurvivors (6, 42%) (Table 2). The median ICU LOS was 4 days for the whole cohort. When compared to survivors, nonsurvivors had a longer LOS in the ICU (median 5 days vs. 4 days, *p* = 0.004) (Table 2). Notably, the main reason for admission for nonsurvivors was renal disease (34.3%), followed by sepsis (31.4%), both of which were more frequent causes of admission than in survivors (11.3%, 6.3%, resp., *p* = 0.002, *p* < 0.001, resp.) (Table 2). The all-cause mortality rate in the ICU was 18.0%.

### 3.2. Characteristics and Outcome of Sepsis Patients

A total of 45 patients (23.2%) in the study cohort were diagnosed by the attending clinicians as having sepsis during their ICU stay. Sepsis was identified on admission to the ICU in 42 patients (93.3%). When comparing the demographics of the sepsis and nonsepsis groups, sepsis patients tended to be older (66.53 ± 13.6 vs. 57.93 ± 16.5, *p* value = 0.002), while no significant differences were found in the gender of the patients, their BMI, or their smoking status (Table 3). Among 11 recorded comorbidities in this study derived from the Charlson Comorbidity Index (CCI) [13], sepsis patients had a higher combined number of comorbidities (2.27 ± 1.51 vs. 1.27 ± 1.09, *p* < 0.001). Furthermore, only 13.3% of patients with sepsis did not have any comorbidity compared to 26.2% of the nonsepsis group. Some comorbidities were significantly overrepresented in sepsis patients, and among those were renal disease (48.9% vs. 12.1%, *p* < 0.001), cerebrovascular diseases, heart failure, and chronic obstructive pulmonary disease (Table 3).

The principle source of admission for sepsis patients was the emergency department (46.7%), and this was followed by hospital wards (33.4%), highlighting the importance of hospital acquired sepsis. The majority of sepsis patients (80%) were managed in the surgical ICUs. Sepsis patients had a longer ICU LOS than nonsepsis patients (median 7 days vs. 4 days, *p* value < 0.001) and a longer hospital stay as well (median 11 days vs. 9 days, *p* = 0.044) (Table 4).

Importantly, the mortality rate was 57.8% in sepsis patients compared to only 6.0% in patients who did not have sepsis (*p* < 0.001) (Table 4). The attending clinicians assigned a diagnosis of septic shock to 33 sepsis patients (77.3%) on the basis of profound circulatory abnormalities despite vasopressor therapy, although lactate levels were not available, and hence one of the Sepsis-3 criteria for septic shock could not be identified. Nevertheless, those patients had a cardiovascular SOFA score ≥3, indicating severe circulatory dysfunction that could explain the high mortality in the sepsis group.

Blood tests of ICU patients on admission were examined. Sepsis patients had higher total WBC count (15.91 ± 7.99 vs. 11.92 ± 5.13 10^9^/L, *p* = 0.001), higher neutrophil counts (8.59 ± 0.67 vs. 7.85 ± 1.29 10^9^/L, *p* < 0.001), and lower lymphocyte counts (0.87 ± 0.56 vs. 1.453 ± 1.03 10^9^/L, *p* < 0.001). Additionally, sepsis patients had higher creatinine (2.40 ± 2.16 vs. 0.89 ± 0.85 mg/dl, *p* < 0.001) and lower haemoglobin values (10.8 ± 2.32 vs. 11.79 ± 2.34 g/dL, *p* = 0.014) (Supplementary Table 2). As for vital signs recorded on admission, sepsis patients had higher temperature and blood pressure readings, while differences in heart rate and respiratory rate did not reach statistical significance (Supplementary Table 2).

### 3.3. Medical Interventions Used for Sepsis Patients

To understand the burden of sepsis in the ICU in terms other than mortality and LOS, we assessed the use of various medical interventions in the sepsis and nonsepsis groups, namely, the use of respiratory support, blood products, and catheters (Table 5).

Patients often had to use more than one respiratory support modality during their ICU stay. On average, each patient with sepsis used more respiratory assistance methods compared to patients who did not have sepsis (2.1 ± 1.49 vs. 1.4 ± 1.02, resp., *p* = 0.002). The use of invasive mechanical ventilation was significantly higher in patients with sepsis (57.8% vs. 8.7%, *p* < 0.001), as was the use of noninvasive mechanical ventilation in the form of CPAP and BiPAP (28.9% vs. 4.0%; *p* = 0.002). Similarly, the use of blood products, measured as a transfusion event of any blood product, was more common in patients with sepsis (60.0% vs. 36.9%; *p* = 0.009) (Table 5).

When investigating the use of arterial and venous catheters, central venous catheters were used more often in sepsis patients (51.1% vs. 26.8%; *p* = 0.003). However, we did not find significant differences in the use of chest tubes (13.3%, 8.7%; *p* = 0.393) or urinary catheters (84.4%, 89.9%; *p* = 0.420), while nasogastric tubes were found to be used less in sepsis patients (48.9%, 65.6%; *p* = 0.012) (Table 5).

### 3.4. Microbiological Characterization of Sepsis Patients

Microbiological culture results were available for 39 sepsis patients; microbial growth was identified in 27 patients (69.2%) (designated as culture-positive sepsis), while the remaining 12 (30.8%) showed no growth in culture (designated as culture-negative sepsis). There was no significant difference between culture-positive or culture-negative sepsis in ICU mortality (55.5% vs. 58.3%, resp., *p* = 0.758) or median ICU LOS (7 days vs. 5.5 days, *p* = 0.192). The suspected origin of infection was designated by the attending clinician as gastrointestinal in the majority of patients (37.8%), followed by the genitourinary tract (24.4%), and the respiratory tract (24.4%) (Table 6).

Among the isolated organisms, we found a predominance of Gram-negative bacteria in 77.1% of all samples that showed any growth, with *Escherichia coli* and *Acinetobacter baumani* each found in 20.8% of the samples (Table 6). While coagulase negative staphylococci were the most commonly isolated Gram-positive species and were found in 20.8% of samples (Table 6).

We also noted the type of sample ordered for culture (e.g., blood, urine, skin swabs, and body fluid) and the rate of positive growth in each type (Table 6). More than one sample were obtained for the majority of patients and were sent for culture with a total of 135 samples. The most ordered sample for culture was blood with 64 samples, 28.1% of which were positive, while the samples that most frequently returned positive microbial growth were soft tissue and skin samples (75%) (Table 6).

## 4. Discussion

This study defined sepsis as life-threatening organ dysfunction associated with infection as proposed by Sepsis-3 [10]. We found no data on sepsis epidemiology among the adult population in Jordan using the aforementioned definition. The definition of sepsis matters in the prognostication of patients since those labeled as having sepsis are expected to have a difficult clinical course and poor outcome compared to those without sepsis, which effectively alters management and resource allocation in the ICU. In general, we found that the Sepsis-3 definition managed to categorise a set of patients who have higher mortality, longer LOS, and more comorbidities and required more frequent medical intervention than nonsepsis patients.

We found that the all-cause ICU mortality rate was 18.0%, which is lower than the mortality rate of 34.6% reported by a retrospective multicentre study conducted in Jordan from 2014 to 2017 [14]. The study noted that university hospitals such as the one in which we conducted this study had lower mortality rates. This supports previous studies showing that ICU specific factors (e.g., organization, staff, and equipment) have a more considerable influence on the patient outcome than regional factors (e.g., ICUs within the same city or country) [15]. On the other hand, the average ICU LOS of around 6 days was concordant with previous reports from Jordan and regionally [1].

We found that 23.2% of patients had sepsis during their ICU stay, which is slightly under the global average of around 29.5% [1]. While all test results required to label a patient as having sepsis according to Sepsis-3 were available (e.g., creatinine, bilirubin), patients' lab profiles lacked a point of care test for lactate. Hence, we could not identify an exact subset of septic shock patients according to Sepsis-3. Sepsis-induced hypotension persisting despite adequate fluid resuscitation diagnosed/identified by clinicians as septic shock was found in 33 of the 45 sepsis patients. This could explain the high mortality rate at 57.8%, a figure around ten times that of nonsepsis patients in this study, and around two times that of sepsis patients in global estimates [1].

Additionally, sepsis patients had a longer median LOS (7 days) compared to patients who did not have sepsis during their ICU stay (4 days), emphasising the prolonged burden among sepsis patients in ICUs. Indeed, this analysis showed that sepsis patients strained ICU resources since they underwent endotracheal intubation and mechanical ventilation, which has been reported to contribute the most to the daily cost of ICU patients [16], almost 5 times that of the nonseptic patients. Moreover, the increased LOS combined with more frequent use of medical equipment in sepsis patients would eventually lead to increased hospital costs and extended stays in the ICU, in concurrence with other reports [6, 17, 18]. This should be taken into consideration when managing limited ICU resources, especially in surge situations such as the current COVID-19 pandemic.

Patients in the ICU often suffer from diseases other than that for which they were admitted to the ICU; hence, we aimed to identify comorbid conditions associated with sepsis. Increased numbers of comorbid conditions have been shown to increase ICU mortality [19, 20]. In this cohort, the combined number of comorbidities was higher in sepsis compared to nonsepsis patients (2.3 ± 1.5 vs. 1.27 ± 1.09, *p* < 0.001), and in nonsurvivors compared to survivors (1.5 ± 1.3 vs. 1.3 ± 1.1, *p* < 0.001). Diabetes mellitus type-2 (DM-2) was the most common comorbidity, found in 47.9% of all ICU patients and in 60.0% of sepsis patients, which is not surprising given the high and increasing prevalence of DM in Jordan [21, 22], and the devastating multisystem effects of DM [23]. The comorbidity that stands out in the sepsis and nonsurvivors' groups was renal disease, which included acute and chronic kidney diseases. Renal diseases were previously shown to be associated with increased organ failure and ICU mortality [24, 25].

When investigating the microbiological results of sepsis patients, causative microorganisms were not isolated in 30.8% of the patients. Notably, the Extended Prevalence of Infection in Intensive Care (EPIC) II study found that 30% of all infections in ICUs worldwide were culture negative [26]. Yet contrary to several reports of increased mortality and LOS in culture-positive sepsis [27, 28], we did not find any significant difference in ICU mortality or LOS with regard to culture positivity. Several factors could have contributed to this result, including sample size, low yield of microorganisms due to prior antibiotic treatment, sample transportation, and the presence of slow-growing and fastidious microorganisms [29, 30].

Some limitations and strengths of this study need to be addressed. First, the ICUs in which we conducted this study are not representative of all ICUs in Jordan, since academic hospitals often provide clinical capabilities unavailable in nonacademic hospitals [14, 31]. Another limitation is the sample size; we were not able to achieve the desired sample size due to changes in the ICUs' structure (done in order to accommodate COVID-19 patients) which eventually led to termination of the study. Nevertheless, this study provided a reference point for future studies in Jordan that will further characterise sepsis patients. Finally, we believe that the prospective data collection performed by the research team on a daily basis provided more accurate, complete, and consistent data when compared to retrieving data from patients' medical records.

In conclusion, this study helped clarify the burden of sepsis in a tertiary academic hospital ICU setting, in a LMIC using updated sepsis definitions. The burden of sepsis in Jordan is high as illustrated by the high mortality, increased LOS, and more frequent use of medical interventions for sepsis patients. The creation of a national sepsis registry in Jordan could guide future ICU policies and would increase awareness of recent sepsis definitions among ICU staff, which should eventually improve patient management. Further studies are needed to assess the burden of sepsis in the ICU in LMIC, and this data could influence international sepsis management guidelines and would guide the decision makers in the allocation of limited equipment and staff to the ICU.

## Figures and Tables

**Table 1:** Demographics and comorbidities of the study cohort divided by outcome.

Characteristic	Entire cohort	Survivors	Nonsurvivors	*p*-value^1^
	*N* = 194	*N* = 159 (82.0%)	*N* = 35 (18.0%)	
Age	59.9 ± 16.30 (62)	58.8 ± 16.60 (61)	65.2 ± 13.60 (67)	**0.049**

*Sex:*				
Male	107 (55.2%)	88 (82.2%)	19 (17.8%)	1.000
Female	87 (44.8%)	71 (81.6%)	16 (18.4%)	

BMI	28.7 ± 6.80 (27.7)	28.5 ± 6.10 (27.6)	29.6 ± 9.30 (27.7)	0.670

*Smoking status:*				
Nonsmoker	117 (60.3%)	95 (81.2%)	22 (18.8%)	0.962
Smoker	54 (27.8%)	45 (83.3%)	9 (16.7%)	
Ex-smoker	23 (11.9%)	19 (82.6%)	4 (17.4%)	

*Comorbidities* ^*2*^ *:*				
Number of comorbidities	1.5 ± 1.3(1)	1.31 ± 1.14(1)	2.34 ± 1.47(2)	<0.001
None	45 (23.2%)	42 (93.3%)	3 (6.7%)	
1-3	133 (68.6%)	110 (82.7%)	23 (17.3%)	
≥4	16 (8.2%)	7 (43.8%)	9 (56.2%)	

*Types:*				
CVD	21 (10.8%)	11 (52.4%)	10 (47.6%)	**0.001**
Dementia	3 (1.5%)	3 (100%)	0 (0%)	1.000
Liver diseases	3 (1.5%)	2 (66.7%)	1 (33.3%)	0.451
Peptic ulcer	4 (2.1%)	4 (100%)	0 (0%)	1.000
Connective tissue diseases	1 (0.5%)	1 (100%)	0 (0%)	1.000
Cancer	43 (22.2%)	33 (76.7%)	10 (23.3%)	0.369
Renal diseases	40 (20.6%)	23 (57.5%)	17 (42.5%)	**<0.001**
IHD	37 (19.1%)	28 (75.7%)	9 (24.3%)	0.340
Heart failure	31 (16.0%)	25 (80.6%)	6 (19.4%)	0.802
Diabetes	93 (47.9%)	71 (76.3%)	22 (23.7%)	0.062
COPD	15 (7.7%)	8 (53.3%)	7 (46.6%)	**0.008**
Hypertension	111 (57.2%)	86 (77.5%)	25 (22.5%)	0.088

All results are presented as count and percent (n (%)) for categorical variables and mean ± SD (median) for continuous variables. ^1^Statistically significant values are shown in bold. ^2^Total >100% since more than one comorbidity or reason for admission were considered for the same patient. Measurement unit: age: years, BMI: kg/m^2^. BMI: body mass index, CVD: cerebrovascular diseases, CTD: connective tissue disease, IHD: ischemic heart disease, and COPD: chronic obstructive pulmonary disease.

**Table 2:** Characteristics related to ICU admission and stay in the study cohort divided by outcome.

Characteristics	Entire cohort	Survivors	Nonsurvivors		*p*-value^1^
	*N* = 194	*N* = 159 (82.0%)	*N* = 35 (18.0%)		
*Service unit:*					
Surgical ICU	124 (63.9%)	103 (83.1%)	21 (16.9%)		0.698
Medical ICU	14 (7.2%)	8 (57.1%)	6 (42.9%)		**0.023**
Anesthesia ICU	43 (22.2%)	37 (86.0%)	6 (14.0%)		0.507
Shared among the three ICUs	13 (6.7%)	11 (84.6%)	2 (15.4%)		1.000

*Admission source:*					
Emergency	82 (42.3%)	69 (84.1%)	13 (15.9%)		0.573
Hospital wards	26 (13.4%)	17 (65.4%)	9 (34.6%)		**0.027**
Operations room	76 (39.2%)	67 (88.2%)	9 (11.8%)		0.086
Dialysis unit	1 (0.5%)	0 (0%)	1 (100%)		0.180
Other	9 (4.6%)	6(66.7%)	3 (33.3%)		0.208

*Reason(s) for admission:*					
Postoperation	79 (40.7%)	69 (87.3%)	10 (12.7%)		0.129
Bowel obstruction	19 (9.8%)	16 (84.2%)	3 (15.8%)		1.000
Due to sepsis	21 (10.8%)	10 (47.6%)	11 (52.4%)		<0.001
Lower limb ischemia	6 (3.1%)	4 (66.7%)	2 (33.3%)		0.296
Renal disease	30 (15.5%)	18 (60%)	12 (40%)		0.002

*LOS in days:*					
Hospital LOS	12.8 ± 13.80 (9)	13.0 ± 14.14 (9)	12.1 ± 12.27 (8)		0.288
ICU LOS	5.8 ± 6.12 (4)	5.3 ± 5.80 (4)	7.9 ± 7.08 (5)		0.004

All results are presented as count and percent (n (%)) for categorical variables and mean ± SD (median) for continuous variables. ^1^Statistically significant values are shown in bold. ICU: intensive care unit, LOS: length of stay.

**Table 3:** Demographics and comorbidities of sepsis and nonsepsis patients.

Characteristics	Sepsis	Nonsepsis	*p*-value^1^
	*N* = 45 (23.2%)	*N* = 149 (76.8%)	
Age at admission	66.5 ± 13.60 (67)	57.9 ± 16.50 (60)	**0.002**

*Sex:*			
Male	24 (53.3%)	83 (55.7%)	0.865
Female	21 (46.7%)	66 (44.3%)	

BMI	29.2 ± 9.10 (27.8)	28.5 ± 5.90 (27.7)	0.978

*Smoking status:*			
Nonsmoker	27 (60%)	90 (60.4%)	0.107
Smoker	9 (20%)	45 (30.2%)	
Ex-smoker	9 (20%)	14 (9.4%)	

*Comorbidities* ^*2*^ *:*			
Number of comorbidities	2.27 ± 1.51(2)	1.27 ± 1.09 (1)	**<0.001**
None	6 (13.3%)	39 (26.2%)	
1-3	28 (62.2%)	105 (70.5%)	
≥4	11(24.4%)	5 (3.4%)	

*Types:*			
CVD	9 (20.0%)	12 (8.1%)	**0.031**
Dementia	0 (0%)	3 (2.0%)	1.000
Liver diseases	1 (2.20%)	2 (1.3%)	0.549
Peptic ulcer	0 (0%)	4 (2.7%)	0.575
CTD	0 (0%)	1 (0.7%)	1.000
Cancer	14 (31.1%)	29 (19.5%)	0.106
Renal diseases	22 (48.9%)	18 (12.1%)	**<0.001**
IHD	10 (22.2%)	27 (18.1%)	0.523
Heart failure	12 (26.7%)	19 (12.8%)	**0.036**
Diabetes	27 (60.0%)	66 (44.3%)	0.088
COPD	7 (15.6%)	8 (5.4%)	**0.049**
Hypertension	29 (64.4%)	82 (55.0%)	0.304

All results are presented as count and percent (n (%)) for categorical variables and mean ± SD (median) for continuous variables. ^1^Statistically significant values are shown in bold. ^2^Total >100% since more than one comorbidity or reason for admission were considered for the same patient. Measurement unit: age: years, BMI: kg/m^2^. BMI: body mass index, CVD: cerebrovascular diseases, CTD: connective tissue disease, IHD: ischemic heart disease, and COPD: chronic obstructive pulmonary disease.

**Table 4:** Characteristics related to ICU admission and stay in sepsis and nonsepsis patients.

Characteristics	Sepsis	Nonsepsis	*p*-value^1^
	*N* = 45 (23.2%)	*N* = 149 (76.8%)	
*Service unit:*			
Surgical ICU	36 (80%)	88 (59.0%)	**0.013**
Medical ICU	6 (13.4%)	8 (5.4%)	0.096
Anesthesia ICU	2 (4.4%)	41(27.5%)	**0.001**
Shared among the three ICUs	1 (2.2%)	12 (8.1%)	0.306

*Source of admission:*			
Emergency	21 (46.7%)	61 (40.9%)	0.497
Hospital wards	15 (33.4%)	11 (7.4%)	**<0.001**
Operations room	6 (13.3%)	70 (47.0%)	**<0.001**
Dialysis unit	1 (2.2%)	0 (0%)	0.232
Other	2 (4.4%)	7 (4.7%)	1.000

*Main reason(s) for admission* ^*2*^ *:*			
Postoperative care	7 (15.6%)	72 (48.3%)	**<0.001**
Bowel obstruction	4 (8.9%)	15 (10.1%)	1.000
Sepsis	21 (46.7%)	0 (0%)	**<0.001**
Lower limb ischemia	3 (6.7%)	3 (2.0%)	0.139
Renal disease	17 (37.8%)	13 (8.7%)	**<0.001**

*ICU outcome:*			
ICU mortality rate	57.8%	6.0%	**<0.001**
ICU LOS in days	9.4 ± 9.37 (7)	4.7 ± 4.17 (4)	**<0.001**
Hospital LOS in days	17.5 ± 20.23 (11)	11.4 ± 10.86 (9)	**0.044**

All results are presented as count and percent (n (%)) for categorical variables and mean ± SD (median) for continuous variables. ^1^Statistically significant values are shown in bold. ICU: intensive care unit, LOS: length of stay.

**Table 5:** Medical interventions used for sepsis and nonsepsis patients.

Medical intervention		Sepsis	Nonsepsis	*p*-value^1^
		*N* = 45 (23.2%)	*N* = 149 (76.8%)	
*Respiratory support:*				
Standard oxygen therapy		30 (66.7%)	120 (80.5%)	0.067
Noninvasive mechanical ventilation (CPAP, BiPAP)		13 (28.9%)	13 (8.7%)	**0.002**
Invasive mechanical ventilation		26 (57.8%)	15 (10.1%)	**<0.001**

*Catheters used:*				
Arterial line	22 (48.9%)		64 (43.0%)	0.498
Peripheral venous line	44 (97.8%)		148 (99.3%)	0.411
Central venous	23 (51.1%)		40 (26.8%)	**0.003**
Chest tube insertion	6 (13.3%)		13 (8.7%)	0.393
Urinary catheter	38 (84.4%)		134 (89.9%)	0.420
Nasogastric tubes	22 (48.9%)		42 (65.6%)	**0.012**
Blood product transfusion	27 (60.0%)		55 (36.9%)	**0.009**

All results are presented as count and percent (*n* (%)). ^1^Statistically significant values are shown in bold. ^2^The total is >100% since one or more interventions were used for the same patient over their ICU stay. CPAP: Continuous Positive Airway Pressure. BiPAP: Bilevel Positive Airway Pressure.

**Table 6:** Microbiological findings in sepsis patients.

^*1*^ *Suspected origin of infection, n (%)*
Gastrointestinal, 17 (37.8%)
Respiratory, 11 (24.4%)
Genitourinary, 11 (24.4%)
Skin and soft tissue, 6 (13.3%)
Others, 16 (17.8%)

^*2*^ *Isolated organisms, n (%)*
Gram-positive bacteria, 17 (35.4%)
Staphylococci (coagulase-negative), 10 (20.8%)
Staphylococci (coagulase-positive), 2 (4.2%)
*Enterococcus* species, 4 (8.3%)
*Streptococcus* species, 1 (2.1%)
Gram-negative bacteria, 37 (77.1%)
*Escherichia coli*, 10 (20.8%)
*Acinetobacter baumani*, 10 (20.8%)
*Klebsiella* species, 9 (18.8%)
*Pseudomonas aeruginosa*, 3 (6.3%)
Others, 5 (10.4%)
Fungi, 10 (20.8%)
*Candida* species, 10 (20.8%)

*Sample type, n (% positive culture growth)*
Blood, 64 (28.1%)
Urine, 38 (28.9%)
Soft tissue and skin, 8 (75.0%)
Catheters and tubes, 6 (50.0%)
Sputum 4, (75.0%)
Pleural fluid and peritoneal fluid, 12 (58.3%)
CSF, 3 (0%)

All results are presented as count and percent (*n* (%)). ^1^More than one origin of infection were suspected in some patients. ^2^More than one pathogen were isolated in some samples.

## Data Availability

All the data analysed during this study are included in this published article.

## Supplementary Materials

Supplementary Materials Supplementary Table 1: vital signs of the study cohort divided by outcome. Supplementary Table 2: vital signs and routine blood tests of sepsis and nonsepsis patients.
