# Supplementary material for: Characteristics of Adult Sepsis Patients in the Intensive Care Units in a Tertiary Hospital in Jordan: An Observational Study
Source: Crit Care Res Pract. 2021 Dec 30;2021:2741271. doi: 10.1155/2021/2741271 (PMC8736695; doi:10.1155/2021/2741271)
Supplement: Supplementary Materials — Supplementary Table 1: vital signs of the study cohort divided by outcome. Supplementary Table 2: vital signs and routine blood tests of sepsis and nonsepsis patients. [file 2741271.f1.docx]

Supplementary material

**Characteristics of adult sepsis patients in the intensive care units in a tertiary hospital in Jordan, an observational study**

**Anas H. A. Abu-Humaidan^1*^, Fatima M. Ahmad^1,2^, Maysaa’ A. Al-Binni^2^, Amjad Bani Hani^3^, Mahmoud Abu Abeeleh^3^**

^1^ Department of Pathology, Microbiology and Forensic Medicine, School of Medicine, The University of Jordan, Amman, Jordan.

^2^ Department of Clinical Sciences, School of Science, The University of Jordan, Amman, Jordan.

^3^ Department of General Surgery, Section of Cardiovascular Surgery, Jordan University Hospital, Amman, Jordan.

*Address correspondence to:

Anas Abu-Humaidan M.D. Ph.D.

E-mail: [A.abuhumaidan@ju.edu.jo](mailto:A.abuhumaidan@ju.edu.jo)

Tel. number: +962779227922

**Supplementary table 1. Vital signs of the study cohort divided by outcome.**

| Characteristic | Entire cohort | Survivors | Non-survivors | P-value^1^ |
| --- | --- | --- | --- | --- |
|  | **N= 194** | **N= 159 (82.0%)** | **N= 35 (18.0%)** |  |
| Vital signs at admission: | | | | |
| Heart rate (beat/minute) | 88.2 ± 18.10 (88) | 87.3 ± 17.25 (88) | 92.4 ± 21.19 (85) | 0.226 |
| Temperature (℃) | 36.7 ± 0.65 (36.7) | 36.7 ± 0.66 (36.6) | 37.0 ± 0.56 (37.0) | **<0.001** |
| Respiratory rate (breaths/minute) | 20.1 ± 4.80 (20) | 20.3 ± 4.65 (20) | 19.2 ± 5.50 (20) | 0.210 |
| Systolic BP (mmHg) | 127.6 ± 21.80(125) | 129.4 ± 20.2(130) | 119.5 ± 26.90 (115) | **0.014** |
| Diastolic BP (mmHg) | 73.9 ± 15.60 (75) | 74.9 ± 15.20 (75) | 68.9 ± 16.80 (65) | **0.036** |
| MAP (mmHg) | 91.6 ± 16.50(92) | 92.7 ± 15.70(93) | 86.3 ± 19.30(82) | **0.037** |

All results are presented as (mean ± SD (median)). ^1^ Statistically significant values are shown in **bold**. Abbreviation: BP; blood pressure, MAP; mean arterial blood pressure.

**Supplementary table 2. Vital signs and routine blood tests of sepsis and non-sepsis patients.**

| Characteristic | Sepsis | | Non-sepsis | P-value^1^ |
| --- | --- | --- | --- | --- |
|  | **N= 45 (23.2%)** | | **N= 149 (76.8%)** |  |
| Vital signs at admission: | | | | |
| Heart rate (beat/minute) | 94.0 ± 22.70 (90) | | 86.4 ± 16.10 (88) | 0.059 |
| Temperature (℃) | 36.9 ± 0.58 (37) | | 36.7± 0.66 (36.6) | **0.013** |
| Respiratory rate (breaths/minute) | 20.4 ± 5.50 (20) | | 19.9 ± 4.60 (20) | 0.761 |
| Systolic BP (mmHg) | 116.5 ± 26.50 (115) | | 130.9 ± 19.02 (130) | **<0.001** |
| Diastolic BP (mmHg) | 65.9 ± 16.10 (65) | | 76.3 ± 14.70 (75) | **<0.001** |
| MAP (mmHg) | 83.2 ± 18.10 (80) | | 94.1 ± 15.20 (93) | **<0.001** |
| Laboratory tests results at admission | |  | |  |
| Haemoglobin, g/dL | 10.8 ± 2.32(10.4) | | 11.79 ± 2.34(12.0) | **0.014** |
| PCV, % | 33.96 ± 6.82(32.9) | | 35.79 ± 6.65(36.3) | 0.110 |
| WBC count 10^9^/L | 15.91 ± 7.99(13.7) | | 11.92 ± 5.13(11.3) | **0.001** |
| Neutrophils (N) 10^9^/L | 8.587 ± 0.67(8.8) | | 7.854 ± 1.29(8.2) | **<0.001** |
| Lymphocytes (L) 10^9^/L | 0.87 ± 0.56(0.8) | | 1.453 ± 1.03(1.1) | **<0.001** |
| N/L ratio | 14.79 ± 10.17(11.9) | | 9.49 ± 8.39(7.2) | **<0.001** |
| Platelets 10^9^/L | 272.58 ± 164.3(240) | | 278.4 ± 126.1(269) | 0.424 |
| Sodium, mmol/L | 136.4 ± 4.9(137) | | 137.7 ± 4.75(138) | 0.201 |
| Potassium, mmol/L | 4.56 ± 0.99(4.3) | | 4.27 ± 0.59(4.3) | 0.202 |
| Chloride, mmol/L | 100.71 ± 7.50(101) | | 102.82 ± 6.04(103) | 0.051 |
| Creatinine, mg/dl | 2.40 ± 2.16(1.7) | | 0.89 ± 0.85(0.65) | **<0.001** |
| RBS, mg/dl | 169.8 ± 95.5(145) | | 166.29 ± 76.02(147) | 0.907 |

All results are presented as (mean ± SD (median)). ^1^ Statistically significant values are shown in **bold**. Abbreviation: BP; blood pressure, MAP; mean arterial blood pressure, PCV; packed cell volume, WBC; white blood cell, RBC; random blood sugar.
